# BCG vaccination at three different age groups: response and effectiveness

**DOI:** 10.1186/1476-8518-3-1

**Published:** 2005-04-01

**Authors:** George Briassoulis, Irene Karabatsou, Vasilis Gogoglou, Athina Tsorva

**Affiliations:** 1 Department of Pediatrics, Markopoulo District Health Centre, Markopoulo Attikis, Greece; 2 Current affiliation Pediatric Intensive Care Unit, University Hospital of Heraklion, Faculty of Medicine, Heraklion, Crete, Greece; 3 Microbiology and Transfusion Departments, NIMTS Hospital, Athens, Greece

## Abstract

**Background:**

The protection, which some BCG vaccines could confer against the development of tuberculosis (TB) in childhood, might be indirectly reflected by the subsequent development of BCG immune response. The objectives of the study were to examine effectiveness and possible differences of post-vaccination reaction to a lyophilized BCG at different age groups and to evaluate its protection against TB in a decade's period.

**Methods:**

We studied the post-vaccination PPD-skin reaction and scar formation at three different school levels, corresponding to ages of 6, 12 and 15 years old, vaccinated by a lyophilized BCG vaccine (Pasteur Institute), currently used in our country. During a 10-year follow up the reported TB cases in vaccinated and non-vaccinated adolescences up to 24-years old were analyzed and compared to the number of cumulative cases observed in the adult population of two neighboring territories (vaccinated and non-vaccinated).

**Results and Discussion:**

There was a significant correlation (r^2 ^= 0.87, p < 0.0001) between tuberculin induration and scar formation. There was no statistically significant difference between the three age groups (6, 12, and 15 year-old, respectively) in regard to the diameter of tuberculin induration or scar formation. Although 34% of 10-year later indurations were unpredictably related to the initial ones (increased or decreased), they were significantly correlated (r^2 ^= 0.45, p = 0.009). The relative percentage of TB for the 14–24 years-age group to the adult studied population was significantly lower among the immunized children compared to the non-immunized population of the same age group (17/77, 22% vs. 71/101, 70%, p < .0001).

**Conclusion:**

Our data suggest that the lyophilized BCG vaccine used for BCG programs at different age groups is equally effective and may confer satisfactory protection against tuberculosis in puberty.

## Background

As one third of the world's population is already infected with mycobacterium tuberculosis (TB), efficacious control of TB, one of the world's major health threats, is best achieved by a combination of chemotherapy and vaccination. Bacillus Calmette-Guerin (BCG) vaccination is compulsory in 64 countries and recommended in others [1]. Recently, the World Health Organization expanded programs of immunization recommended BCG at 3 months [2], while in many areas there is vaccination at birth [3], at school entry and in adolescence [4]. The policy in Greece for some years has been to recommend BCG vaccination routinely in schools to children aged 11 to 13 years, but adjusted to a current tuberculous infection index of 3.4%, this schedule has been recently endorsed by offering the vaccine at school entry (ages 5 to 7 years), until the risk of infection is low everywhere in Greece. The justification for continuing vaccination throughout the country even although TB is now rarely encountered in some areas, is related to the mobility of the population: many young people study, join the army, or seek work in areas where TB is more prevalent [5].

The efficacy of BCG vaccination, however, has been strongly questioned [6]. Studies in older children and adults showed 77% protection in Britain [7]), only 14% in southern USA [8], and none in Madras [9]. Furthermore, in a retrospective study of 22 children with TB of the spine in a developing country, all gave a history of BCG vaccination scars [11]. In addition, several reports recommending the continuation of the policy of BCG vaccination offered routinely in schools, are now concerned about the influence of the quality of the vaccine, its transportation, and the technique of its application on the protection obtained [10]. Laxity in the TB control programmes and widespread of HIV could also appear to have a role in the recent reurgence of TB infection worldwide [11]. However, methodological and statistical reappraisal has showed that different biological and environmental conditions in individual studies and, principally, biases or inadequate statistical power may have contributed to the conflicting data [12]. Recently, findings at 15 years showed that even among populations with high infection rates and high non-specific sensitivity where BCG did not offer any protection against adult forms of bacillary pulmonary tuberculosis, BCG offered some level of overall protection (up to 50%) in children [13].

BCG induced tuberculin sensitivity is a quantitative characteristic and has been used to compare vaccine efficacy. It has been also suggested that the protection which some BCG vaccines could confer against the development of TB in childhood, might be indirectly reflected by the subsequent development of BCG immune response [14,15]. The preliminary research, however, has been restricted to examine the effectiveness of freeze dried vaccines only, and has not been extended to study the immunologic properties of the lyophilized BCG vaccines, currently used in Europe. Furthermore, those limited studies were properly oriented to evaluate the protective effect of BCG in either infant Asians or high risk neonates, thus including too few details to permit assessment of the main clinical expressions of acquired immunity and to draw the indisputable conclusions about the significance and its derived implications among the general population.

As part of a prospective assessment of the efficacy of a lyophilized BCG vaccine used in current BCG schemes in Greece, we had the opportunity to evaluate its immunologic response to study the interrelations between the post-vaccination tuberculin sensitivity and scar formation, and to analyze their epidemiological data in children who had received BCG in three different age groups. During the next decade, we comparatively recorded the 10-year reported TB cases in vaccinated and non-vaccinated adolescents up to 24-years old in our territory and in a neighboring area not covered by similar preventive program, and compared them to the 10-year number of cumulative cases observed in the adult population of the two areas.

## Methods

### Participants

Intradermal BCG vaccination has been offered routinely to schoolchildren living in the district area covered by our health center since 1988 and continues up to date. Children participating in the preventive program from 1/10/1988 to 1/10/1993 were enrolled in the study. To restrict escapes, BCG scheme was annually applied at three different school levels, corresponding to ages of about 6, 12 and 15 years old, respectively. All children monitored in each of the cohorts were documented to be receiving their first BCG vaccination when they were in their respective age groups and had not previously been vaccinated (there is no neonatal BCG program in Greece and also all children should have had negative tuberculin testing before been enrolled in the study). Children, who had received BCG vaccination at birth or at any other time because of known contact with a case of TB, were excluded from the study. Likewise, notified subjects given chemoprophylaxis, whether tuberculin positive or negative, were also excluded from analysis. Of a total of 1,124 vaccinated schoolchildren included in the final analysis (group A), 394 received their first vaccination at age 6 and accordingly were classified in the 6 year -old group, 483 in the 12 year-old first vaccination group and 247 in the 15 year-old first vaccination group.

### Methodology

Vaccine was given by a doctor and team of health visitors experienced in vaccination technique. Lyophilized vaccine (0,1 ml, Pasteur inst.) was injected intra-dermaly over the insertion of the left deltoid muscle to produce a weal of about 7-mm diameter, using a separate syringe and a 27-G needle for each person [10]. The reconstituted vaccine used at our center contains about 0.15 mg moist weight of Galmette-Guerin organisms per ml, which implies a concentration of colony-forming units of viable organisms of 6 × 10^6^/ml. In all cases using 10 UI of PPD was performed three days before vaccination along with a thorough clinical examination. Tuberculin testing was repeated three months after vaccination when children were again reviewed. The result was read three days later and induration of 6 mm or more, when read across the forearm, was regarded as positive result [14]; induration of 4–5.9 mm as a weak positive and induration less than 4 mm as a PPD-negative. Scar formation was classified as real scar (>2 mm), tiny (≤ 2 mm), or not visible [10]. No other immunologic studies were done to correlate immunity to BCG with assessment of other in vitro responses to vaccination, since in such a massive program it would not be feasible to do many things in many individuals in a short time, especially, in a school environment. As the study did not affect patient care, the institutional review board waived the need for informed parental consent.

### Sub cohorts

Those who gave a negative result retested within one month. In proved tuberculin negative responders (Mantoux < 4 mm), revaccination was suggested and if consent was obtained, the vaccine was given apart from the site of the scar of the previous BCG vaccination (group R) as it was previously advised [10].

### Controls

Children in schools not participating in the BCG prophylaxis program (schools in neighboring areas not covered by our health center) were used as controls for the follow up period (non vaccinated adolescences). In total 1340 control children of comparative age groups were recorded the same time period. The two territories had similar population according to the latest data (2001) available by the National Statistics Center (vaccinated 375024 and non-vaccinated 348236).

### Follow-up

Tuberculin testing was repeated ten years after vaccination in a small proportion of children, still attending school and if an informed parental consent had previously been obtained. Also, during the intervened decade, we comparatively recorded the 10-year reported TB cases in vaccinated and non-vaccinated adolescents from more than 14 up to 24-years old in the two territories. Data were drawn from the Health Centre's records and the National Statistics Center of Infectious Diseases, and subsequently were analyzed and compared to the 10-year number of cumulative cases observed in the adult population (more than 25 years old) in the same areas (vaccination area covered by our health center and non-vaccinated neighboring area not covered by our health center). There were not any epidemiologic data available regarding the incidence of TB in the 6 up to 14-year age group.

### Statistical analysis

Methods for assessing data significance included the two-sample null hypothesis for a two-tailed test, the Mann-Whitney U test and the x^2 ^test with Yates' correction, using a standard statistical package.

## Results

### Tolerability

In all subjects tuberculin testing before vaccination gave a negative result and on the basis of history or clinical examination there was no contraindication to perform BCG vaccination. The procedure was well tolerated with no cases of osteopathy and only very few of reactions such as, three cases of transient lymphadenopathy and one of local subcutaneous abscess.

### Age groups

**Figure 1:**
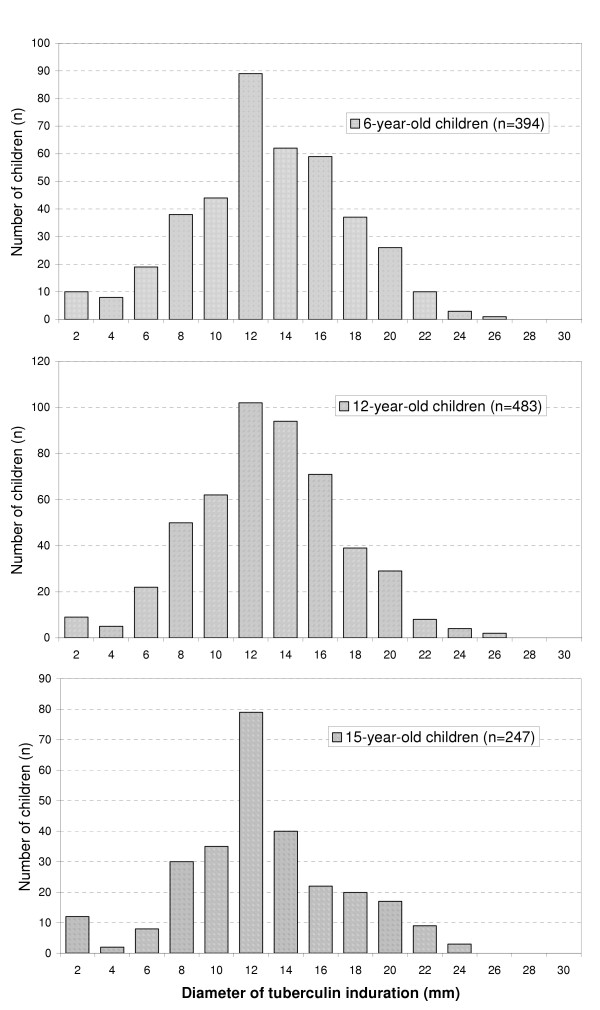
Post-vaccination tuberculin testing in three school-age groups

**Figure 2:**
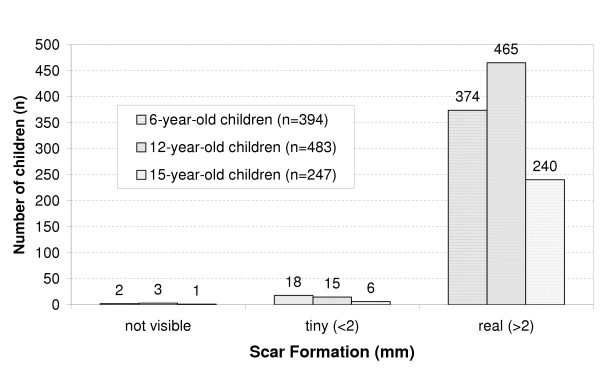
BCG scar formation in three school-age groups

Details of tuberculin testing and scar formation at each age group are shown in Figures 1 and 2. There was no statistically significant difference between the three age groups (6, 12, and 15 year-old, respectively) in regard to the diameter of tuberculin induration or scar formation. The mean (SD) diameter of the tuberculin reaction was 11.13 (4.20) mm. The distribution of Mantoux induration diameters appeared to be the one of normal curve with the 95% confidence limits set at 2.73 mm and 19.53 mm (binomial distribution). Extensive search revealed no evidence of active TB in those schoolchildren with a tuberculin induration greater than or equal to 20 mm. Although 1034 (92.2%) subjects gave a positive post-vaccination tuberculin reaction, 45 (4%) gave a weak positive induration, and 43 (3.8%) gave a PPD-negative test. Real scar formation has been developed in 96% of the vaccinated schoolchildren and a tiny scar (≤ 2 mm) in 3.5%.

### Tuberculin induration and scar formation

**Figure 3:**
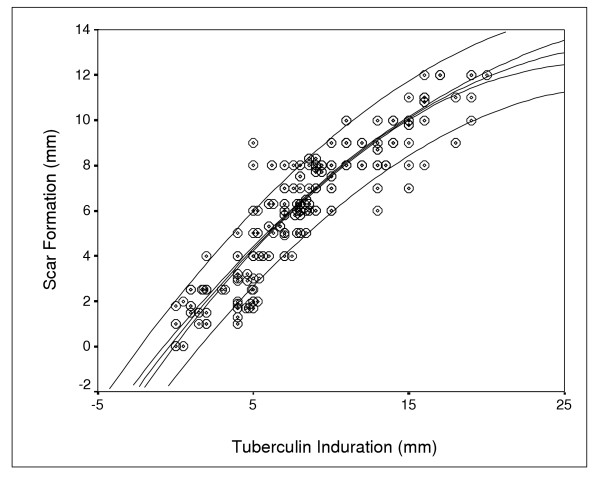
Correlation between tuberculin induration and scar formation

There was a significant correlation (r^2 ^= 0.87, p < 0.0001) between tuberculin induration and scar formation (Figure 3). Table 1 shows that none of the children without scar formation has developed PPD-positive reaction. Instead, 99.4% of the children with positive tuberculin skin testing exhibited scar formation compared to only 39.5% of those who were negative for tuberculin reactivity (p < 0.001). Accordingly, the possibility for a schoolchild to not develop a visible scar has been significantly higher among Mantoux negative children than among those who presented with either a positive Mantoux (p < 0.001), or a weak positive tuberculin testing (p < 0.001). The real estimated percentage of the possibility for absence of scar formation in Mantoux negative children has been included between the 95% limits of 14% and 38%, the most possible value been an estimated percentage of 23 % (binomial distribution).

**Table 1:** No (%) of BCG scar formations in three groups of different post vaccination tuberculin reactions among school children

**Mantoux**	**Children**	**Evaluation of scar formation [n (%)]**		
**Induration (mm)**	**n**	**Real scar (>2 mm)**	**Tiny scar (≤ 2 mm)**	**No scar (?)**
**Negative (0–3,9)**	43	17 (39.5)*	20 (46.5)**,***	6 (14)***,***
**Weak positive (4–5,9)**	45	32 (71.1)	13 (28.9)**,***	0 (0)***,***
**Positive (>6)**	1036	1030 (99.4)*	6 (0.6)***,***	0 (0)***,***

**Total**	1124	1079 (96.0)	39 (3.5)	6 (0.5)

*p *values apply to differences among negative, weak positive and positive indurations for the same scar formation groups (real scar, tiny or not visible scar).

* < 0.01, ** p < 0.025, *** p < 0.001.

Tuberculin negative subjects were more likely to develop a tiny scar than the subjects with a positive (p < 0.001) or weak positive Mantoux test (p < 0.025), respectively. There has been a higher percentage of tiny scars in children with tuberculin indurations of 4–5,9 mm than in those with the next range (6–9 mm) of induration diameters (p < 0.001). The real estimated percentage for the presence of a tiny scar in children with a tuberculin reaction of less than 6 mm has been included between the 95% limits 42% to 54% (mean 47%, binomial distribution)]

### Negative reactors

Of the 43 tuberculin negative reactors, 32 cases were reviewed. Lack of reactivity was confirmed and all 32 had the BCG vaccine repeated. Surprisingly, following revaccination, reactivity levels to 10 UI of PPD (90.6%) were similar to the reactivity rates documented in the initial vaccination group A cohort (92.2%, NS), corresponding to 100% scar formation. There was not only an apparently similar distribution of tuberculin indurations (p < 0.42), but also a similar mean between groups (group R 12.76 (5.40) mm vs. 11.13 (4.20) mm for group A, p = 0.1).

### Follow-up

#### A. Tuberculin induration

**Figure 4:**
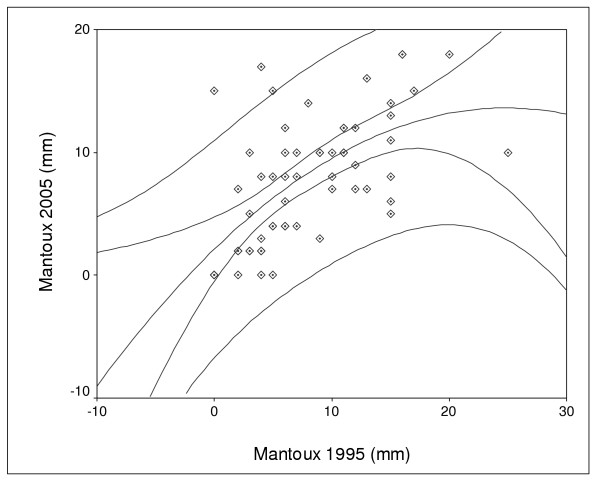
Paired sample correlation (quadratic regression) of tuberculin indurations between the two time – periods (initial study period and 10-year later follow up)

**Figure 5:**
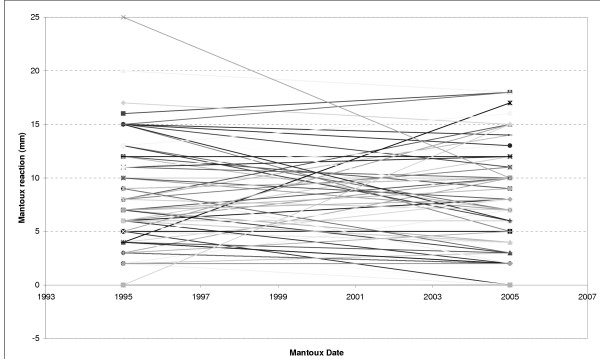
Follow up of recent individual indurations (2005) correlated to initial Mantoux values (10-year time interval)

Ten years later the cohorts became inconsistent and many children disappeared (studies, army, new families) or – when found – most denied having a repeated Mantoux test. Among 183 16-year old schoolchildren (former 6 year -old group) been visited by health visitors at schools in 2005, 110 denied to participate in the follow-up of the study. Thus, approximately 10 years later (2005), we only managed to repeat Mantoux in 73 children of the 6 year-old group of the 1993–5 year period. There was no statistically significant difference between the two year periods in regard to the diameter of tuberculin indurations (mean (SD) diameter of the tuberculin reaction 8.4 (5) mm in 2005 vs. 8.1 (6) mm in 1995). The mean paired difference of the paired samples test was non significant: .25 (6) mm (95% confidence interval of the difference: lower: -1.9, upper: 2.4 mm). Although the paired sample correlation (Figure 4) was significant (r^2 ^.45, p = 0.009) and new individual values were almost identical to the old ones in 66% of children (± 2 mm), a great proportion of the 10-year later values were unpredictably related (12% decreased, 22% increased) to the initial ones (Figure 5).

#### B. TB cases

Data available in the National Statistics Center of Infectious Diseases and in the Health Centre's records showed that the TB cases recorded among vaccinated adolescents – more than 14 up to 24 years-old – during the last decade in our territory were lower compared to the non-vaccinated adolescents (17 vs. 71 cases, in a decade). These numbers would have resulted in the wrong estimation of 1.3% vs. 6.3% TB incidence in adolescenthood for the vaccinated schoolchildren and controls, p < 0.0001, if they had only been restricted to the studied population. However, the recorded TB cases were extended to a decade's population, including at least three times more similarly treated children in each group, making therefore statistical analysis impossible. The numbers of the recorded TB cases for the adult population (>25 years old) in the two territories recorded during the same period were slightly lower for the vaccinated area compared to non-vaccinated territory (77 vs. 101 cases). Thus, the relative percentage of TB for the 14–24 years-age group to the responded adult population, which could provide us with the only comparable numbers, was significantly lower among the immunized children (17/77*100) compared to the non-immunized population (71/101*100) of the same age group (22% vs. 70% of all ages, x^2 ^= 14.7, p < 0.0001).

## Discussion

Because of the conflicting results of major controlled trials, BCG vaccination against TB remains controversial despite more than 50 years of use. The wide range of BCG protective efficacy values reported by the trials extended from 75%, reflecting substantial protection by the vaccine [3] to negative ones indicating a higher rate of TB among vaccinees than controls [16].

Although the tuberculin state after vaccination was not generally thought to influence the degree of protection offered by BCG, children who did not become sensitive to tuberculin either died subsequently of disseminated TB [17] or developed tuberculous meningitis [18]. Furthermore, the relative increase of TB in the mainly unimmunized cohorts born in a European country after 1975, compared with the mainly BCG immunized cohorts born there in the period 1969–1974 was, by the end of 1984, estimated at 6 (95% confidence interval, 2.3 to 16.1) [19]. Subsequently, when a high level of post-vaccination immune response was achieved, the estimated protective efficacy for the vaccine was shown to be 64% with 95% confidence limits of 43% and 77% and the prevented fraction 0.50 [15].

Conflicting results from two centers in the United Kingdom showed that only 45–46% of vaccinated children were Mantoux positive when tested between the ages of 3 months and 2 years [20]; 25% had no visible scar [21]. Another study carried out in a third center, however, showed that 353 (98%) out of 361 Asian neonates given BCG were tuberculin positive when tested three months later [14]. Tuberculin conversion rates of 93% or 88% were also established following BCG vaccine in 15 term and 8 pre-term infants, respectively [22]. In addition, a more recent study among 193 Asian vaccinees revealed a positive post-vaccination tuberculin testing in 184 of them (95%) [15]; these results are consistent with those of the present study confirming a 94% prevalence of immune response to BCG in a sample of 1124 vaccinated Greek schoolchildren. If impaired immunity was the cause of diminished tuberculin sensitivity after vaccination, then a fairly constant proportion of Asian or Greek children would be expected to be non-reactors to tuberculin after vaccination. In another study, significantly higher proportions of infants given Japanese BCG were found to be tuberculin convertors (74.7%) when compared to those given British BCG (51.4%). In our study groups there was no difference between initially or repeatedly vaccinated children (92% vs. 90%), and this was also reflected in the frequency of non-visible scar formations. In the negative reactors, however, BCG vaccination could have triggered protective (Lister type) rather than antagonistic (tuberculin or Koch type) reactions, which have been speculated to be the most protective [23]. Similar to other study findings done in infants [24], we also did not observe any significant difference in mean tuberculin reaction, tuberculin positivity and mean scar size according to different age group at administration.

Results of our study confirm a highly positive correlation between scar formation and post vaccination tuberculin sensitivity among schoolchildren at various ages. This finding is remarkably similar to that suggested by reports in India and the United States, which have shown that the size of the BCG scar was associated with considerable enhancement in sensitization to tuberculin [25,26]. Similarly, in a recent study the prevalence of skin test positivity was also consistently higher among individuals compared to those without a BCG scar [27]. Our data confirm this trend, since children who failed to produce a BCG scar did not have evidence of a positive post-vaccination immune response to tuberculin skin testing. It's important to note however that negative skin test reactivity can still be associated with real (46.5%) or tiny scar (39.5%) formation (37 or 43 patients, total 86%). It must be also noticed that in contrast to most previous studies, this study uses a lyophilized vaccine, demonstrating the basic characteristics of a recently used BCG vaccination in a western country upon the entry of the new millennium.

The 1079 subjects with a scar are assumed to have mounted an initial response to the vaccination, as reflected by a strong positive immune response in the 1030 of them (99.4%). Possibly, a few negative or weak responses were due to physiological implications such as general health nutritional status, prevalence and especially virulence of atypical mycobacteria [23], an infection with *M. Kansasii *approximating 80% of the potential protection offered by BCG [28]. Furthermore, in a population with a low level of sensitivity to PPD (median skin reactions 3 mm), 36% of the subjects gave a greater response to an atypical antigen such as PPD-B [29]. It is unlikely, however, that all negative results were caused by this, since most of the Mantoux negative children turned to become positive reactors after they subsequently underwent repeat BCG vaccination. This finding which has been further enhanced by an observed similar to the expected, according to our results, distribution of tuberculin indurations, is in agreement with recent reports, suggesting that revaccination resulted in a significant increase in positivity to tuberculin 10 and to other reagents tested [30].

Accordingly, it seems unlikely that the impaired reaction to tuberculin in some of the recently vaccinated subjects had been caused by a defect of initial recognition of the antigen or an inability to retain this information or even by a failure of sensitized lymphocytes to react because the individual was malnourished or had a serious infection [23]. Instead, there is circumstantial evidence that even when given at birth, BCG achieves tuberculin conversion in a high proportion of neonates [31], irrespective of race [32], ethnic origin [33] or prematurity [24]. Since age has not been a significant factor in influencing BCG immune response in the present study, the age mass vaccine must be offered at should be ideally adjusted to the one, shortly before the infection rate is going to be accelerated [6].

Although the mean (SD) diameter of postvaccination tuberculin reactors in our study was unexpectedly high, (11.13 (4.20) mm), this was significantly lower than a mean of 17.9 mm calculated for naturally infected subjects [33,34] and was not significantly different from the mean postvaccination reaction of 9.4 (2.7) mm reported by others [15]. Surprisingly, also, a much wider distribution range of tuberculin indurations has been included between the 95% confidence limits for the sample (2.73–19.53 mm). In fact, a 71.1% of those with a weak positive Mantoux (4–5.9 mm) exhibited a scar of >2 mm, which has been previously suggested to adequately represent a positive immune response to BCG vaccination [35]. The rest of them (28.9%) exhibited a tiny BCG scar, presumably underlining a rather smoother and wider transition zone between PPD positive and negative reactions. Examining two different batches of new tuberculin, Stanford JL and Tala-Heikkila found that the mean size of the BCG scar was 8.1 (SD 4.8) mm, and there was a trend associating smaller BCG scars with smaller tuberculin responses, which did not reach statistical significance [36]. Although such an explanation for the right end of the curve appears to be in disagreement with the assumption that reactions larger than 10 mm in diameter are likely to represent infection [30], a similar wide transition zone for variable positive reactions is also strongly supported in our study by the demonstration of a previous negative PPD testing, immediately followed by the BCG vaccination which has been shortly preceded the tuberculin inversion. Thus, the large tuberculin reaction achieved in a substantial proportion of children shortly after BCG might simply represent a strong cell-mediated immune response among individualized expressions of recently acquired immunity. Besides, BCG vaccination at birth and for school age children causes reactivity to tuberculin which persists for 20 to 25 years, so that an induration diameter of > 15 mm does not exclude a vaccinal origin [37]. Similarly, in our study among schoolchildren, no significant waning of immunity to BCG was shown 10 years after initial vaccination. We also showed that although the immune response to BCG persisted for so long, individual responses varied widely (increased or decreased) in 1/3 of children, the meaning of which couldn't be extrapolated. In a recent study, prior BCG vaccination had a strong influence on skin test results of <or = 18 mm in diameter among persons <40 years old, compared with the influence of factors predictive of *M. tuberculosis *infection [38]. For vaccinated subjects with a previous negative tuberculin test, it is also necessary to exclude the booster effect. Thus, according to our results and those of others [39], an induration diameter of > 15 mm does not exclude a vaccinal origin [38]. Additionally, in some cases, such a reaction might also suggest that sensitization to mycobacteria species might have occurred at a very young age. Although such a sensitization might not be detected by the tuberculin test, it can influence response to BCG vaccination [40]. The marked difference of tuberculin reaction rate between Indian towns strongly supports such an influence of exposure to mycobacteria in the environment [27]. Although it has been found that in a population with a high level of sensitivity to PPD (median skin reaction 12 mm), only 7% of the subjects tested gave a greater response to an atypical antigen than to PPD [31], determination of the optimum range of reactions to the BCG vaccination in a given population is a more contentious issue, which cannot be fully resolved without further information about the interactions among the quality of vaccine, the time elapsed, the prevalence and virulence of atypical mycobacteria, the booster effect, the immunologic memory and the mechanisms of its responsiveness to other reagents [40]. It is therefore not possible in the late post-vaccination period to distinguish between a tuberculin reaction caused by virulent supra-infection and one resulted from persistent post-vaccination sensitivity, even in the case of a strong positive reaction to 10 UI of tuberculin PPD.

Repeat BCG vaccination, malnutrition, and BCG with scars present difficulties in making a diagnosis of TB but did not affect PPD reactivity and did highlight the need for thorough clinical evaluation [41]. Although, high tuberculin sensitivity in healthy schoolchildren may be partially maintained by contact with environmental mycobacteria, attributing a 'positive' Mantoux response to past BCG vaccination may be encouraging a false sense of security in contacts recently exposed to an infectious case of TB. Our results confirm the general assumption that the major disadvantage of BCG is that it clouds interpretation of the tuberculin skin test [42]. These results, however, do also suggest that the notification of post-vaccination tuberculin induration diameter about 3 months after BCG, might well be served as a self-control measurement in the case of clinical diagnosis of TB [43], since post-vaccination skin reactions tend usually to decrease with time. Additional factors, such as age of the contact and sputum status of the index case are important determinants of the degree of increased tuberculin sensitivity [44]. However, at the rise of the millennium, new blood assays (QuantiFERON-TB, CSL Limited), which measure gamma interferon production when M. tuberculosis-specific proteins, such as the ESAT-6, are incubated with venous blood samples, are promising for the recognition of infection with Mycobacterium tuberculosis, since they are not influenced by past BCG exposure [45].

Although vaccination does not prevent the establishment of infection in someone exposed to tubercle bacilli, its effect does limit the multiplication and dissemination of tubercle bacilli and the development of lesions after infection. The direct effect of BCG vaccination is defined as prevention of TB in vaccinated persons and the indirect as the reduction of TB in the population as a whole [30]. Results of epidemiological research laboratories, however, showed considerable variation in the current BCG vaccination policy in different districts while the population is highly mobile [37]. To allow the proportion of unvaccinated young people to increase while more than 3,420 new sputum positive cases of TB are reported annually in Greece – now much more increased among AIDS victims -increases the risk of disease, which may well not be diagnosed until others have been infected [46].

Since there is evidence in the neonatal period, but not in childhood, challenging the view that sensitization is essential for protection [47], the assumption that a positive skin test following vaccination is an indicator of BCG-induced immunity against Mycobacterium tuberculosis might not have been a correct one. We thought, however, that because a high rate of PPD skin test conversion following BCG vaccination in schoolchildren and its persistent reactivity for 10 years might both increase the degree of protection offered by BCG, it should be concluded that the particular lyophilized BCG vaccine used for BCG programs in Greek schools may confer satisfactory protection against TB in puberty. Our results are further enhanced by the findings of a recent study, which showed that BCG vaccination at birth and for school age children causes reactivity to tuberculin which persists for 20 to 25 years [48]. The recorded decline of TB among the adolescents in the vaccinated area, highlights the need to sustain and, wherever possible, intensify preventive and case finding measures in all groups at special risk [49], but it might also suggest that the continuation of offering mass BCG vaccination should ideally be maintained everywhere, including regions that currently have a low prevalence of TB [5]. Similarly, in view of the current incidence of TB in Finland and the likelihood that lymph node infections and sensitivity to environmental mycobacteria will increase, continued BCG vaccination at birth has been recommended [37].

One of the main limitations of this study is that the sample size for a comparison of disease occurrence between the study and control groups was too small and thus statistically underpowered for such an analysis. Furthermore, it wouldn't be practically possible to follow up and record disease occurrence in the cohorts, since there was no computerised system importing data from hospitals or insurance agencies into the health centre and patients do not often reveal such medical confidentialities by themselves. Accordingly, since we had covered all schools at three different age-levels in our territory, and there was not any BCG program running in the control territory, we made the logical assumption that vaccination was widespread in our territory, and absent in the other. A severe limitation of this hypothesis, however, is the prerequisite of a second assumption that no significant migration and no significant differences in the prevalence of latent TB infection or the proportion of adult cases with positive sputum smear, must have occurred over the study period. For all these reasons, the prevention part of this study is not so strong and has accordingly not been the primary endpoint of the study. Another limitation of the study is that we did not succeed in following all cohorts further, retesting them some years later to assess age-related differences that may have been substantial in terms of waning responses. Children of the 12 and 15 year-old cohorts had finished school, the cohorts became inconsistent, and even when personal data were still available (addresses, phones) it was found extremely difficult to bring people back to the health centre. The response of the retested patients, however, might offer a realistic idea of what reaction could have been found in the other age groups as well.

Continuation of the BCG programs could easily work as a bridge to the coming era of new generation of TB vaccines. It has been already shown that vaccination with ESAT-6 antigen from mycobacterium tuberculosis, which is a dominant target for cell-mediated immunity in the early phase of tuberculosis, delivered in a combination of monophosphoryl lipid A and dimethyl dioctadecylammonium bromide, which are adjuvant efficients for the induction of cellular and humoral immune responses, elicited a strong ESAT-6-specific T-cell response and protective immunity comparable to that achieved with mycobacterium bovis BCG [50]. As one third of the world's population is already infected with Mycobacterium tuberculosis and because the acquired immune response is mediated by different T-cell sets, two types of vaccine may be required: one for eradication of already established infection and the other for prompt combat of invading microbes [51]. Meanwhile, emphasis should especially be placed on the importance of the quality, transportation and preservation of the vaccine and on the technique of application [10], that may be responsible for the widely divergent results of prospective BCG trials in several countries, that have lead to current doubts about the efficacy of BCG vaccination in TB prevention.

## Competing interests

The author(s) declare that they have no competing interests.

## Authors' contributions

GB conceived of the study, participated in its design, and drafted the manuscript. IK participated in the collection of the data and performed the statistical analysis. VG collection of the data and coordination and helped to draft the manuscript. AT participated in the design of the study and performed the statistical analysis. All authors read and approved the final manuscript.
